# Supplementary material for: Combining LOPIT with differential ultracentrifugation for high-resolution spatial proteomics
Source: Nat Commun. 2019 Jan 18;10:331. doi: 10.1038/s41467-018-08191-w (PMC6338729; doi:10.1038/s41467-018-08191-w)
Supplement: Supplementary file 1 — Supplementary Information [file 41467_2018_8191_MOESM1_ESM.docx]

**Supplementary Information**

Geladaki *et al.*, Combining LOPIT with differential ultracentrifugation for high-resolution spatial proteomics

**
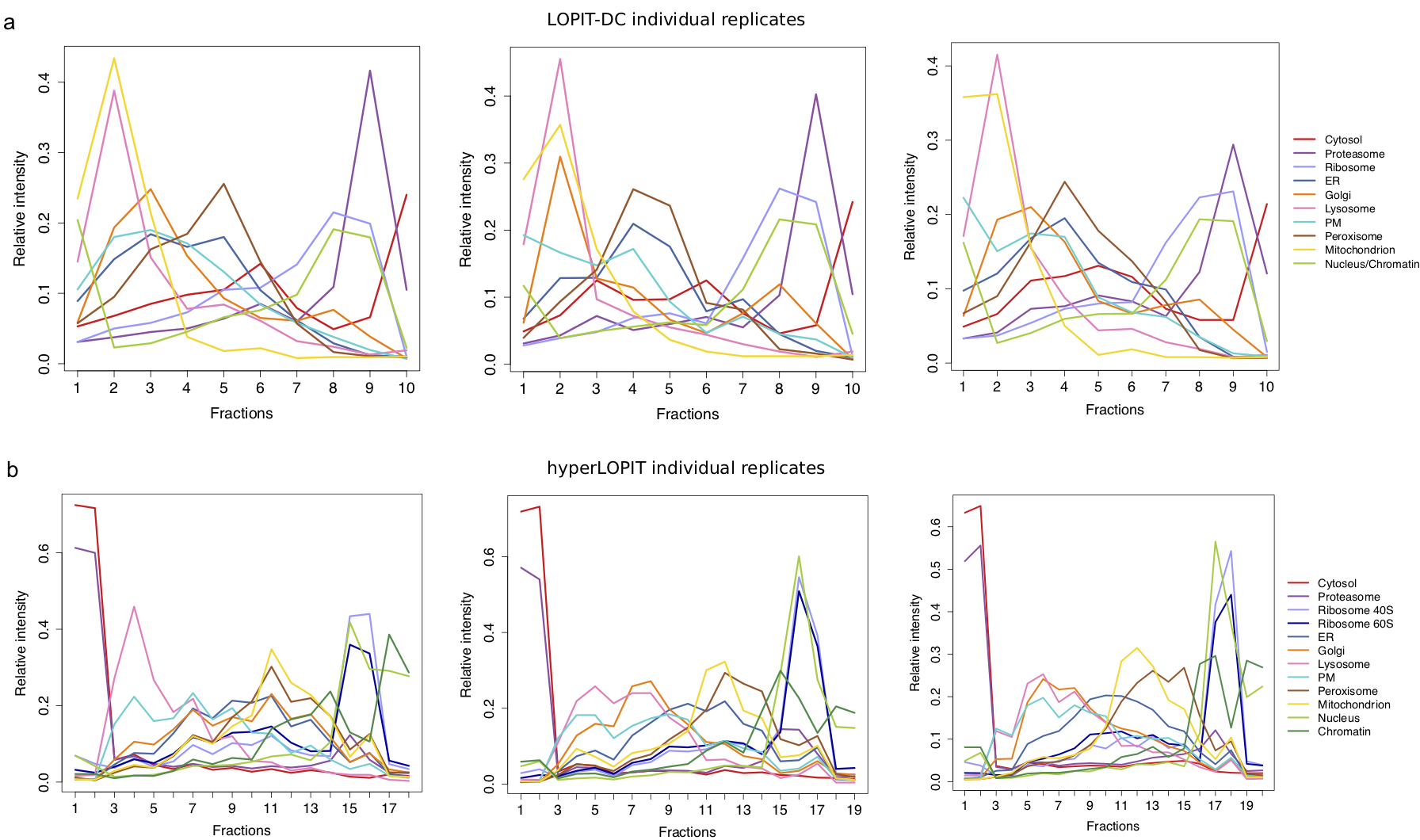
Supplementary Figures**

**Supplementary Figure 1 |** Median organelle marker profiles (TMT reporter ion distributions) in individual replicates for the (a) LOPIT-DC and (b) hyperLOPIT experiments.

The marker profiles in the LOPIT-DC data indicate that organelles sediment at different centrifugation speeds. The first pellets contain larger organelles such as the mitochondria. As suggested by the signal of the ER and Golgi markers, these organelles sediment during centrifugation steps 2-5 with the ER showing a much wider enrichment pattern than the Golgi. The final supernatant is a very concentrated soluble fraction enriched for the cytosol of the cells. The nucleus sediments in the first as well as the final two fractions indicating that this organelle may be partially shredded during differential centrifugation-based subcellular fractionation experiments. The hyperLOPIT marker profiles reveal differential organelle enrichment throughout the density gradient. The low density end of our linear iodixanol gradients is occupied by light organelles whereas the high density end of the gradients is enriched with dense subcellular structures. Indeed, the plasma membrane, lysosomes and Golgi occupy fractions 1-10 while heavier organelles such as the mitochondria and nucleus populate fractions 11-19. Furthermore, the ER is mostly enriched towards the middle, forming a wider distribution than other subcellular particles. Finally, the cytosol is absent from most subcellular fractions and only a weak cytosolic signal can be detected in our first few low density samples, indicating successful separation of the intracellular membranes from the cytosol. In both cases, all subcellular niches show variably overlapping but individually unique enrichment distributions showcasing the high fractionation quality and superior resolution produced by both LOPIT-DC and hyperLOPIT.


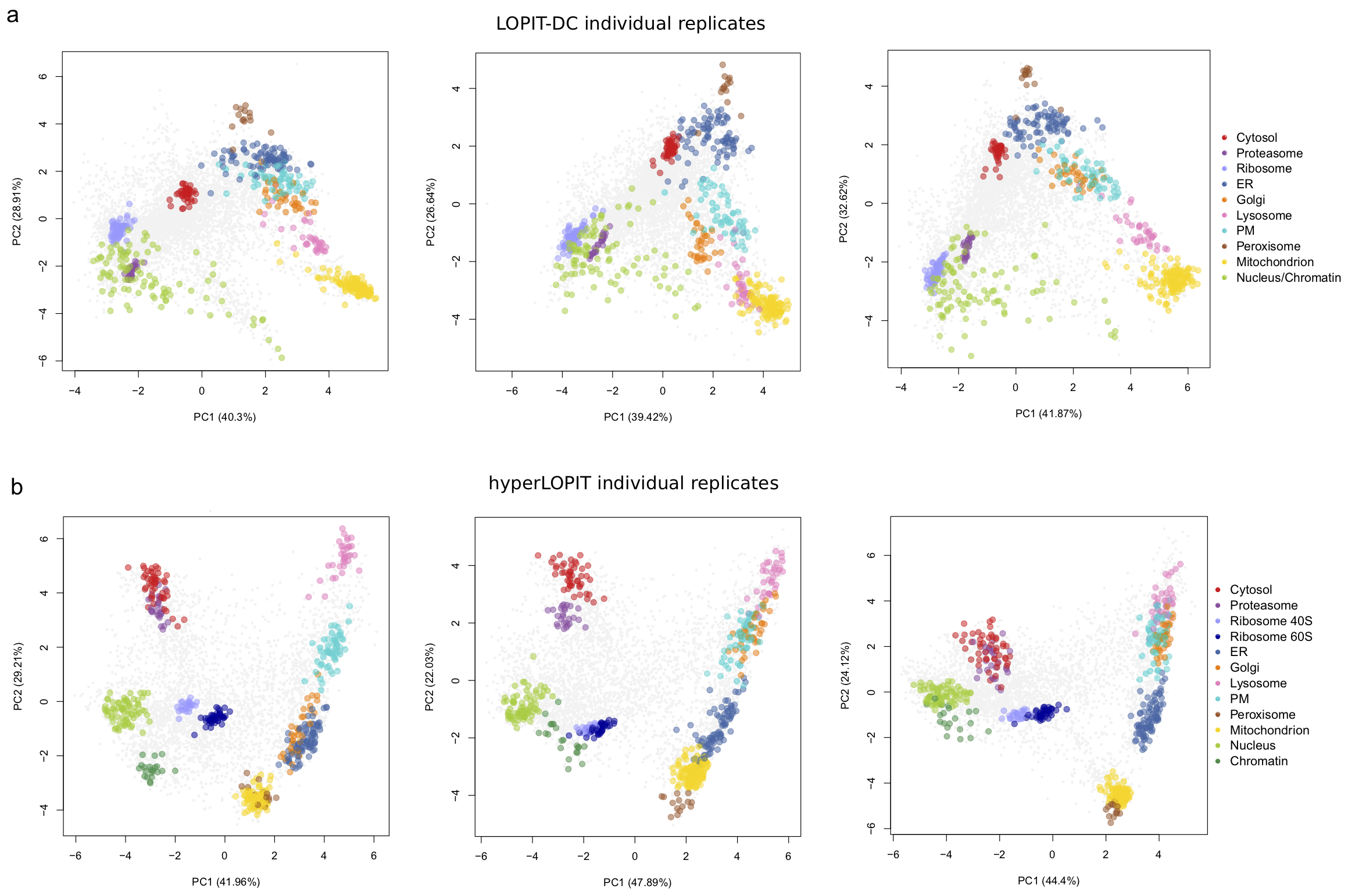


**Supplementary Figure 2 |** PCA plots of individual replicates of the (a) LOPIT-DC and (b) hyperLOPIT experiments.

Based on Principal Components 1 and 2, LOPIT-DC replicate 1 is best at separating the nucleus/chromatin, ribosomes and proteasome away from the rest of the membrane-bound organelles. The cytosol also shows a unique distribution in this replicate, forming a tight cluster further away from all other subcellular niches. Replicate 2 is best at resolving the PM from the ER and Golgi while replicate 3 performs best in separating the lysosome from the mitochondrion and PM/Golgi clusters. HyperLOPIT replicate 1 is best at resolving the PM from the Golgi and lysosome, the chromatin from the non-chromatin nucleus and the two ribosomal subunits from each other. However, in this replicate the distributions of the peroxisome and ER partially overlap with those of the mitochondrion and Golgi, respectively. Our second and third hyperLOPIT replicates were performed with slightly modified centrifugation settings (rotor and duration of equilibrium density gradient ultracentrifugation) in order to achieve separation of the organelles whose distributions were overlapping in replicate 1. Indeed, replicates 2 and 3 succeeded at resolving the peroxisome from the mitochondrion and the ER from the Golgi. Replicate 2 displays additional separation between the proteasomal and cytosolic clusters which is a unique feature of this experiment. This result provides evidence that our cell fractionation pipelines can be adjusted in order to achieve different levels of subcellular resolution.

**
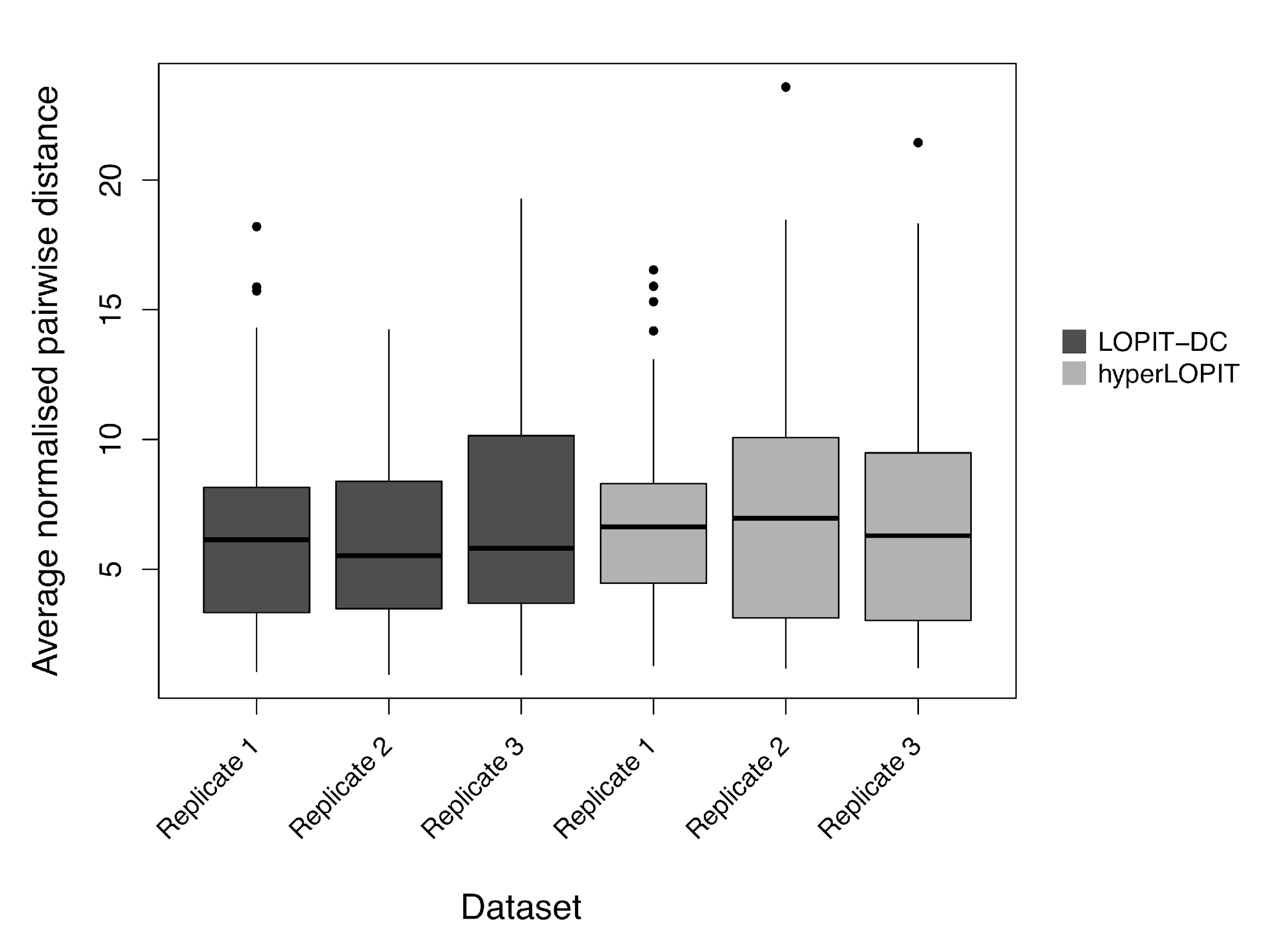
**

**Supplementary Figure 3 |** Boxplots displaying average normalised pairwise distances, as calculated by QSep, for all replicates of the LOPIT-DC and hyperLOPIT datasets.

For each boxplot, the line in the middle of the box is the median value, the vertical size of the box represents the interquartile range (IQR) and the whiskers represent the extremes of the data (defined as those that do not exceed 1.5 x IQR from the middle of the data, and if no points exceed that distance, then the whiskers are simply the minimum and maximum values).


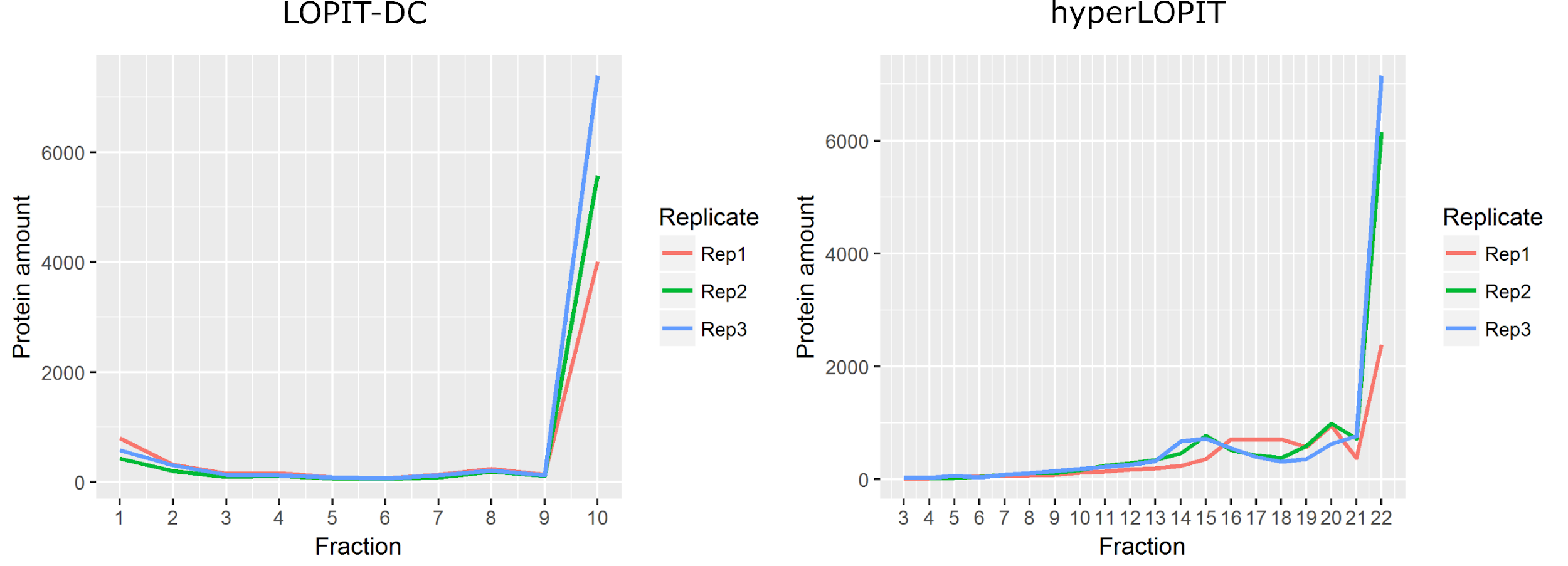


**Supplementary Figure 4 |** Protein yield in the different LOPIT-DC and hyperLOPIT fractions for all replicates.

In the LOPIT-DC data, the final cytosol-enriched supernatant contains the highest protein yield. Larger organelles pellet sooner (see Supplementary Figure 1a) and so the first and second pellets contain the next highest protein amounts. Pellets 5 and 6 contain the lowest amounts of protein. In the hyperLOPIT data, the supernatant not loaded on the iodixanol gradient contains the highest protein yield, similarly to the LOPIT-DC dataset. The low-density fractions recovered from the top of our gradients yielded a relatively low amount of total protein while the middle and high density samples recovered from the middle and bottom of the gradients yielded high protein amounts. This observation is due to the fact that the top of a density gradient is populated with light organelles that are small, low in protein content or present in low numbers in a cell such as the plasma membrane. Conversely, the bottom of a density gradient is occupied by heavy subcellular structures which are usually large, high in protein content or quite abundant in a cell such as the nucleus and mitochondria (see Supplementary Figure 1b).

**
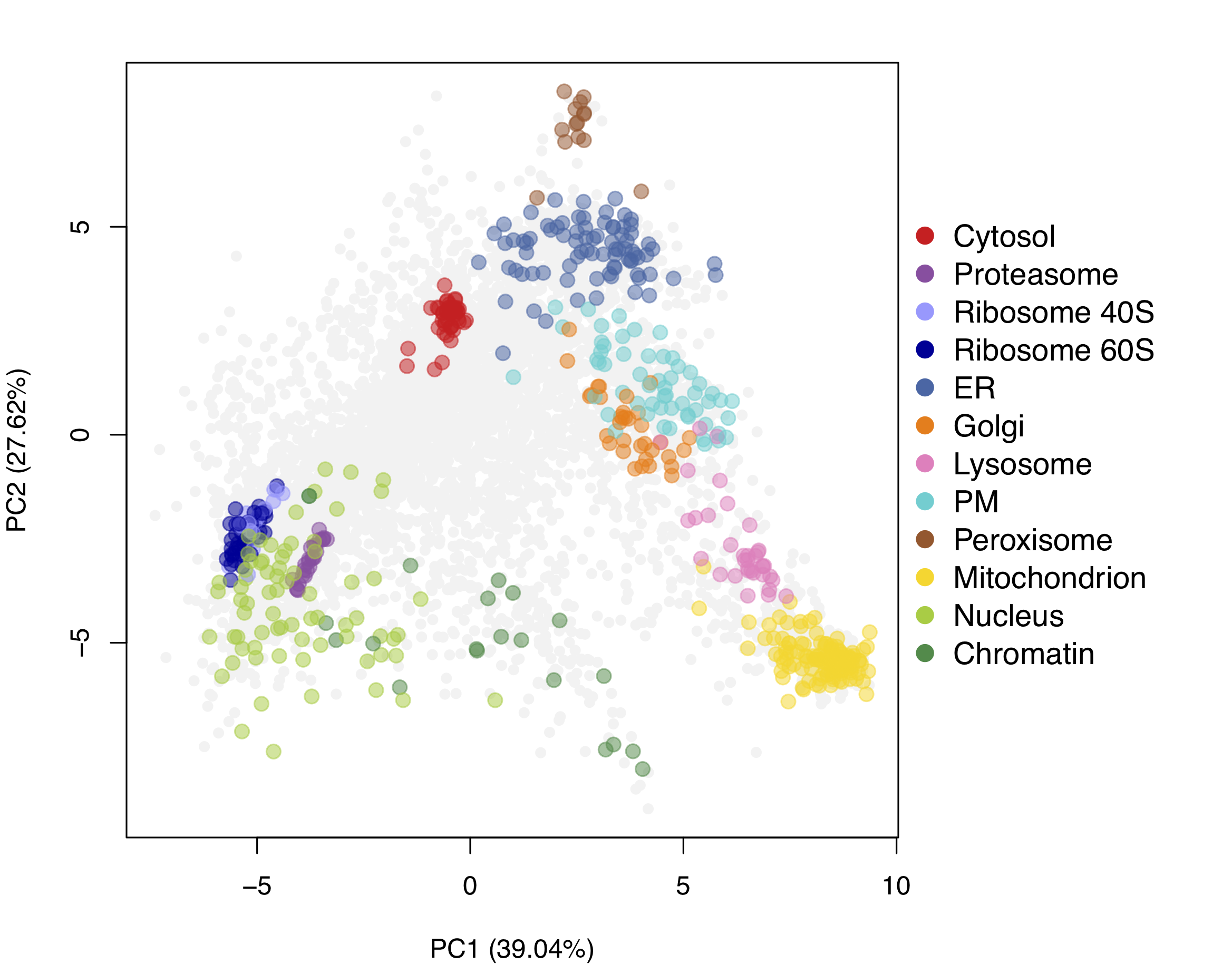
**

**Supplementary Figure 5 |** PCA plot of the merged LOPIT-DC dataset annotated with 12 marker classes.

We find that the ribosome 40S and 60S subunits are not well-separated from each other in our LOPIT-DC data and the same is true for the nucleus and chromatin clusters, motivating our choice to merge these classes for downstream analysis.


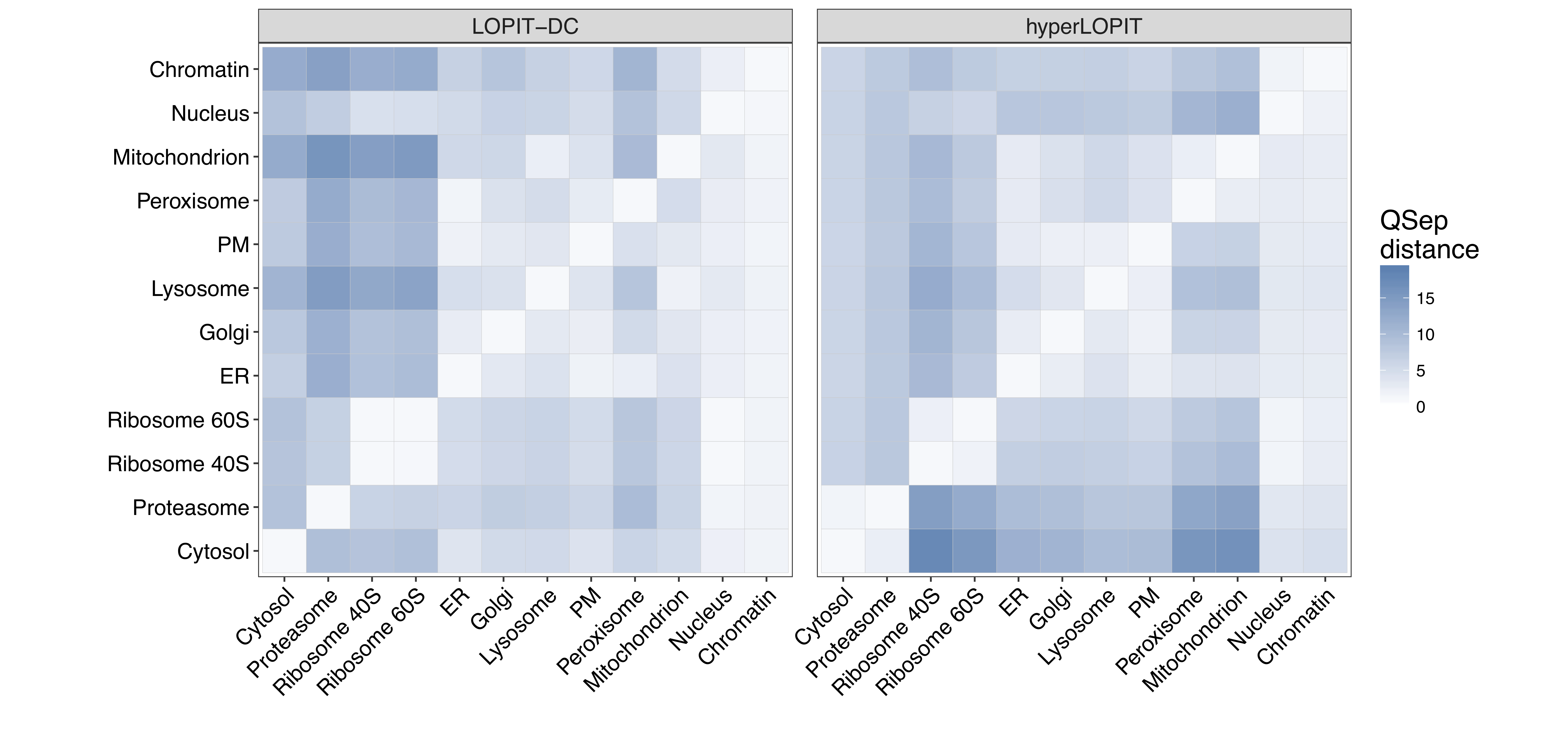


**Supplementary Figure 6 |** QSep distances between all pairs of compartments in the LOPIT-DC (left) and hyperLOPIT (right) datasets, where 12 organelle classes were used to define both datasets.

**
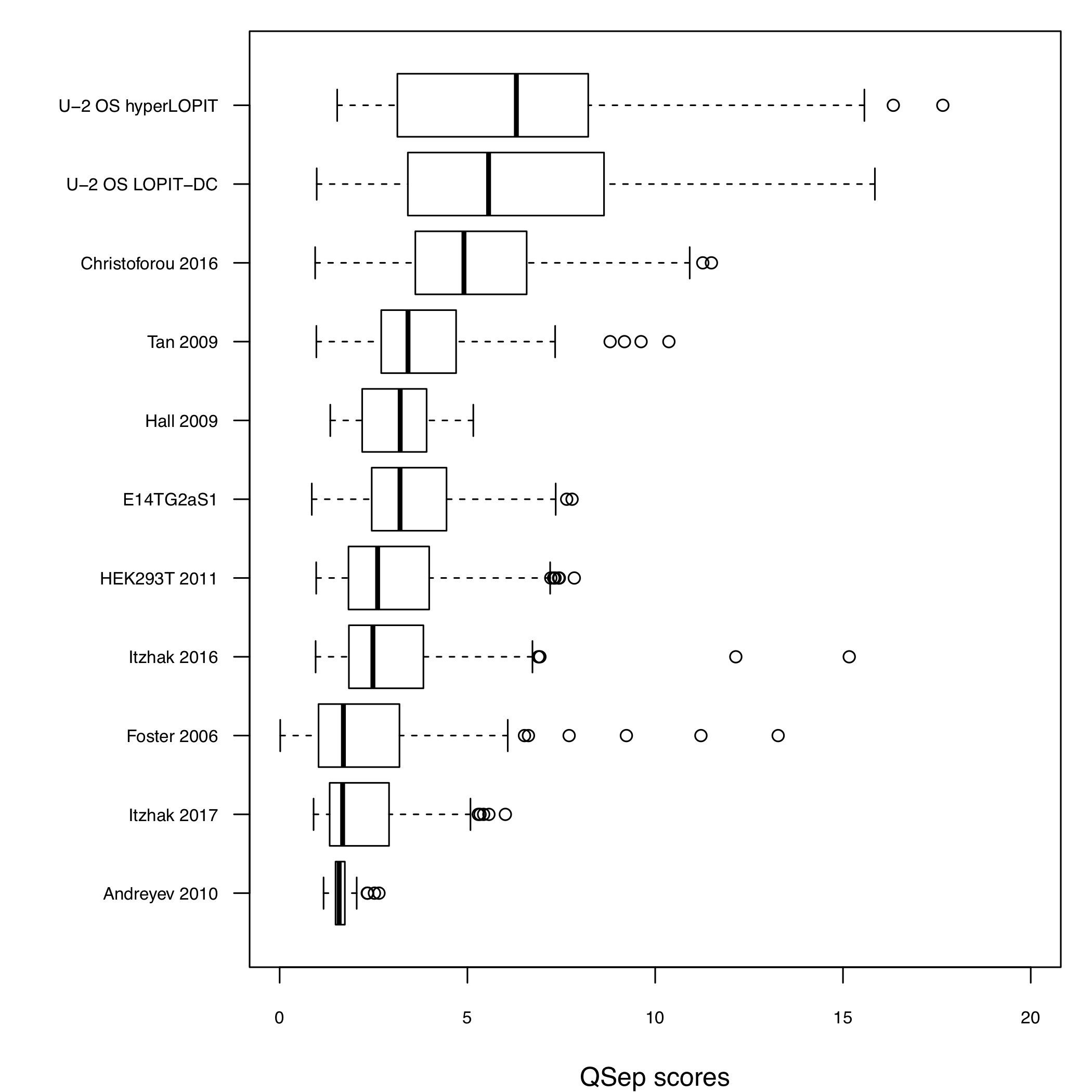
**

**Supplementary Figure 7 |** Boxplots displaying average normalised pairwise distances as calculated using QSep on recent spatial proteomics studies.

For each boxplot, the line in the middle of the box is the median value, the vertical size of the box represents the interquartile range (IQR) and the whiskers represent the extremes of the data (defined as those that do not exceed 1.5 x IQR from the middle of the data, and if no points exceed that distance, then the whiskers are simply the minimum and maximum values).


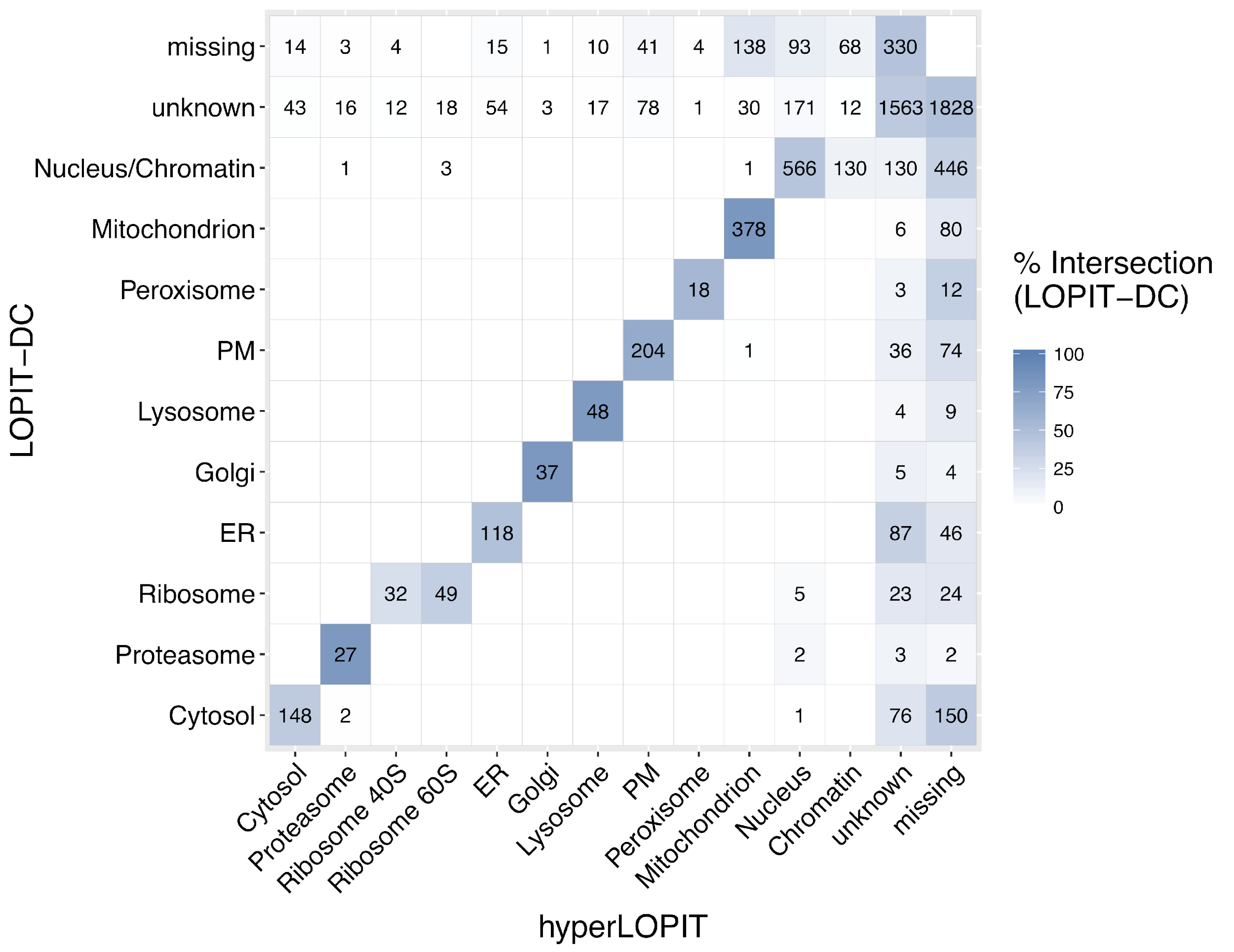


**Supplementary Figure 8 |** Heatmap displaying the overlap between the LOPIT-DC and hyperLOPIT protein subcellular localisation assignments including markers and missing proteins (proteins only present in one dataset but not the other), where the colour code is based on the percentage of intersection (i.e. the number of intersecting proteins is divided by the total number of proteins assigned to that organelle in the LOPIT-DC data).

**
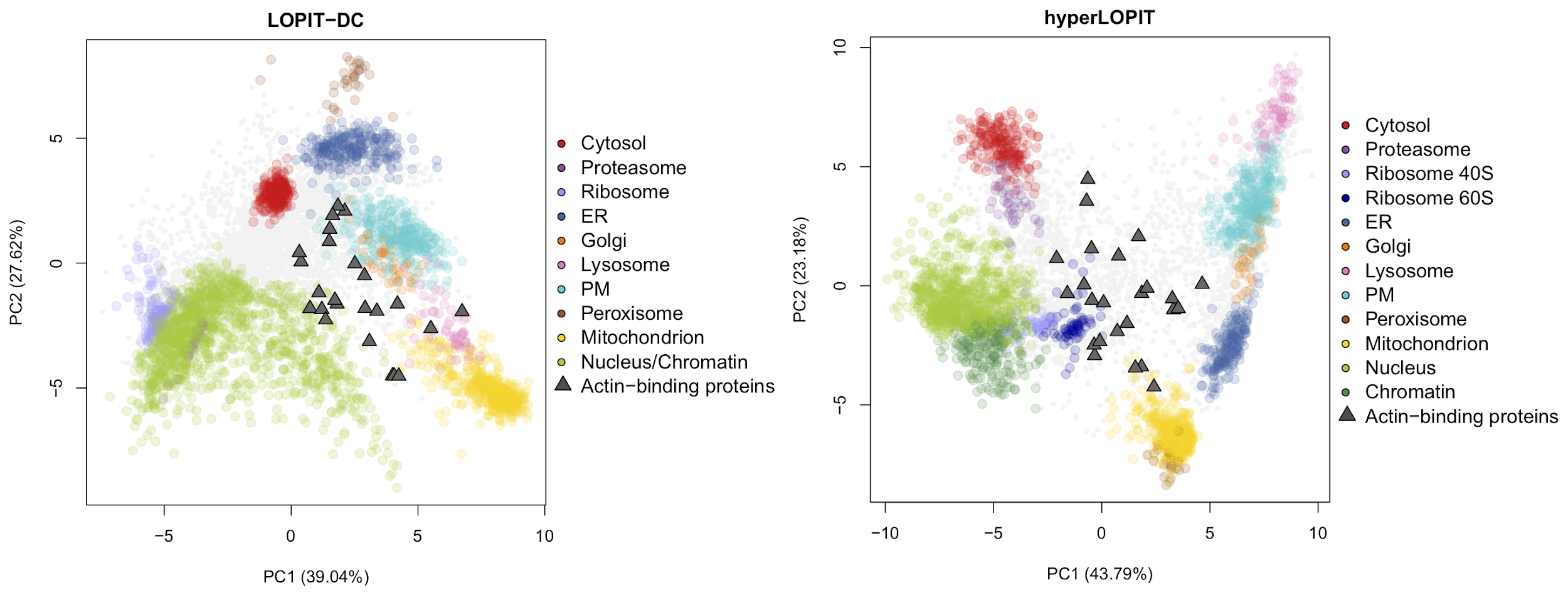
**

**Supplementary Figure 9 |** Actin-binding proteins plotted upon the LOPIT-DC and hyperLOPIT datasets with assigned proteins.


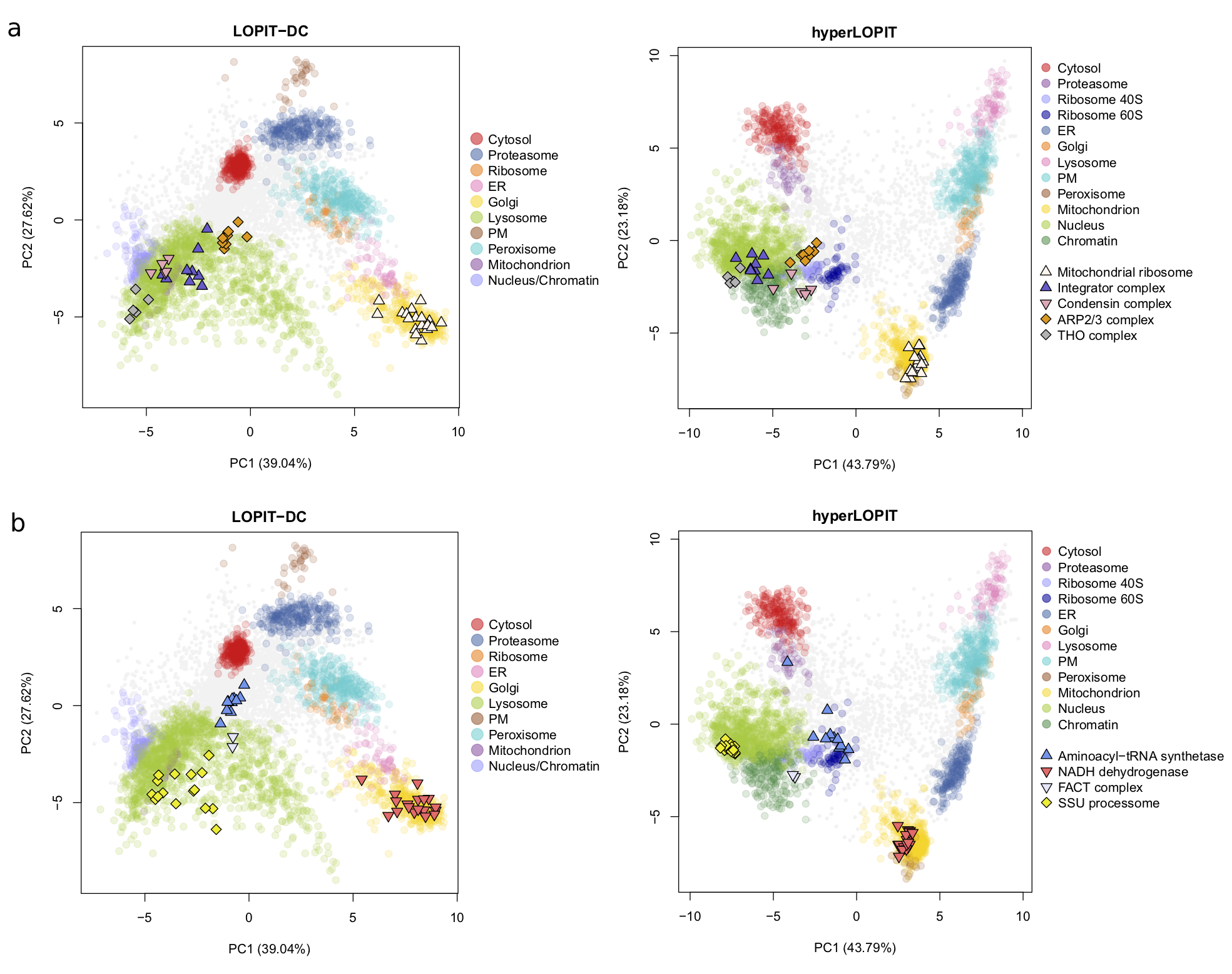


**Supplementary Figure 10 |** Additional protein complexes plotted upon the LOPIT-DC (left) and hyperLOPIT (right) datasets showing the (a) mitochondrial ribosome, integrator complex, condensin complex, ARP2/3 complex, THO complex and (b) aminoacyl-tRNA synthetase, NADH dehydrogenase, FACT complex, SSU processome.

**
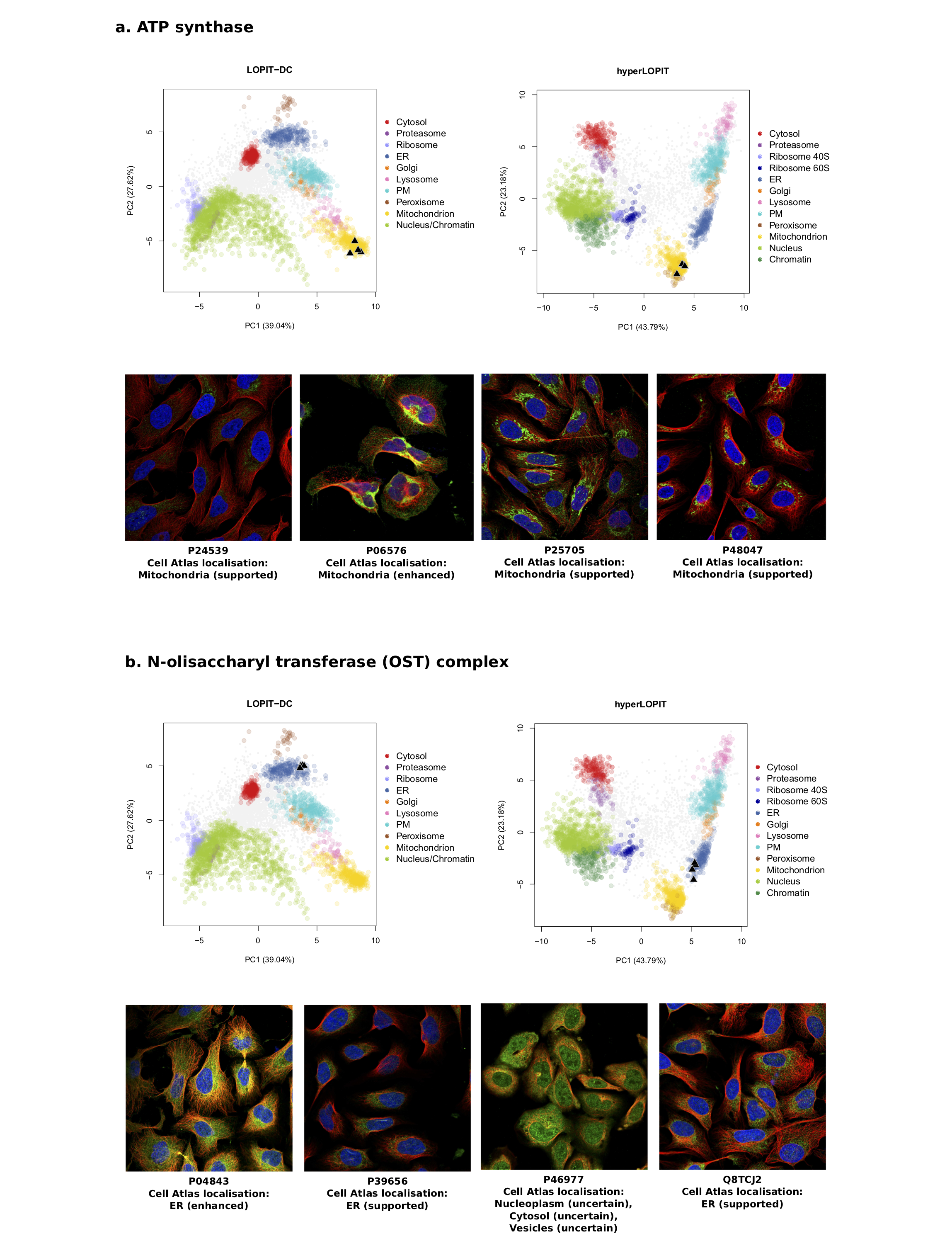
**

**
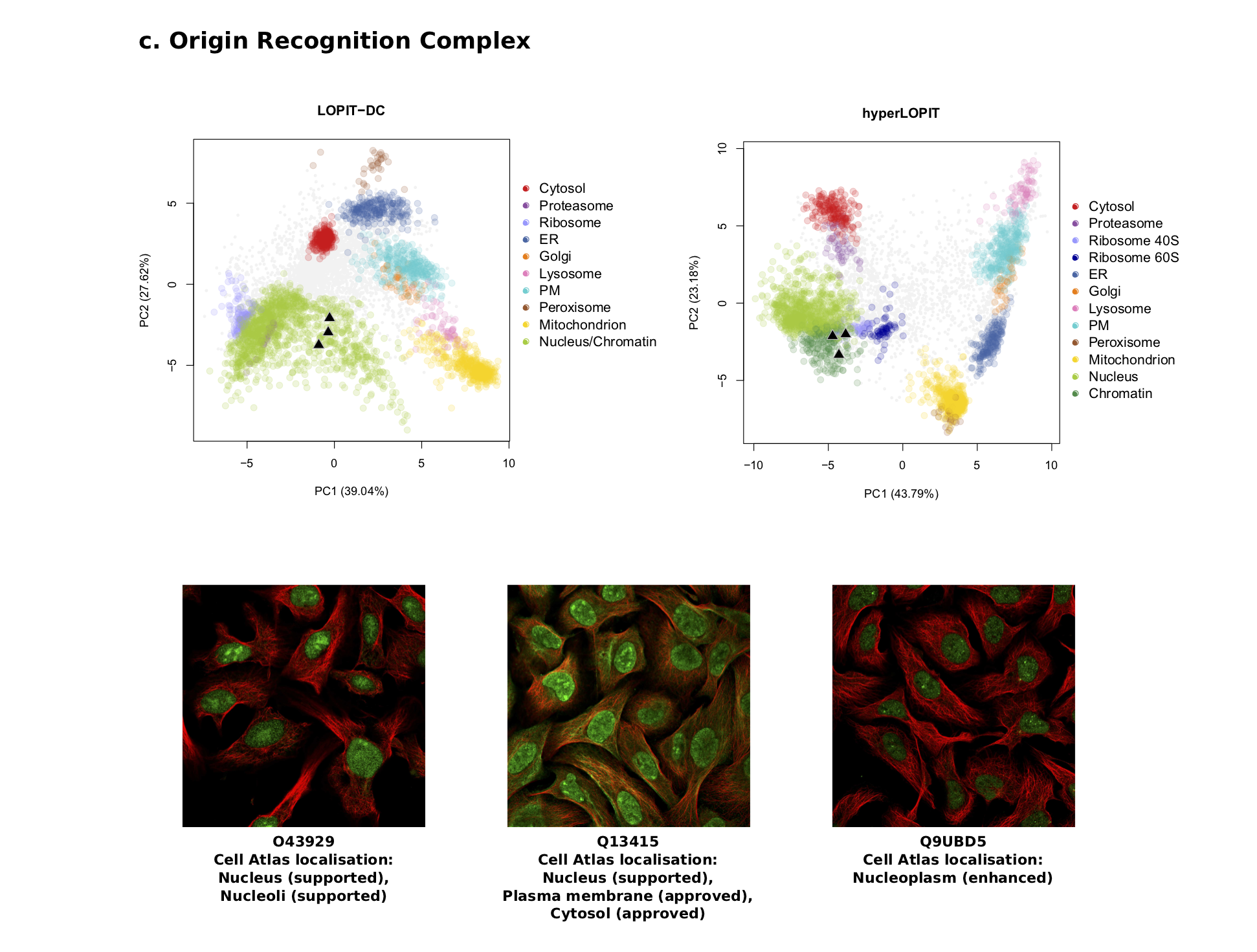
**

**
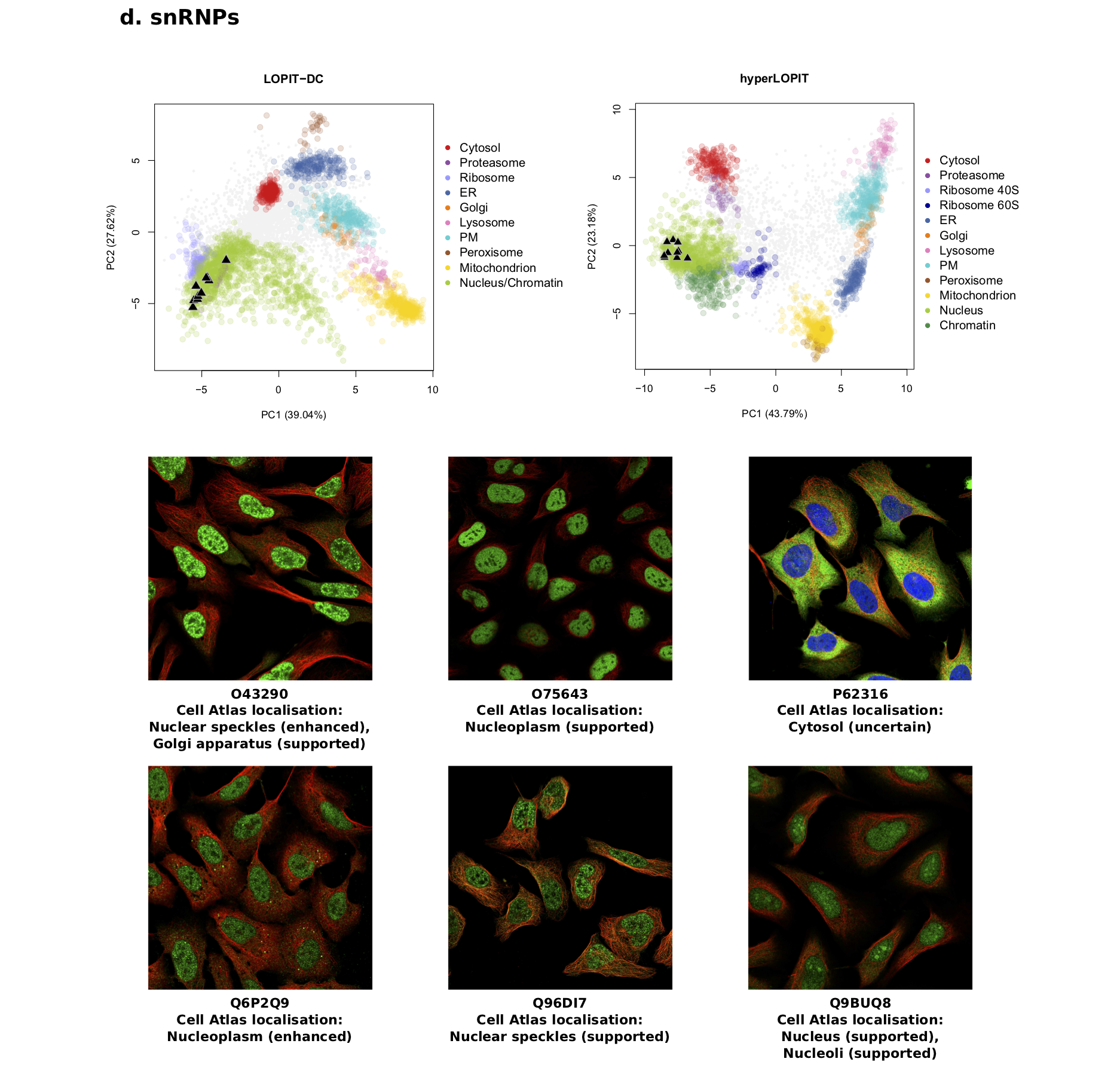
**

**Supplementary Figure 11 |** PCA plots of the LOPIT-DC and hyperLOPIT datasets as well as U-2 OS cell-specific immunofluorescence images and subcellular localisation annotation taken from the Cell Atlas database ([https://www.proteinatlas.org/humanproteome/cell)] for individual proteins which belong to the (a) ATP synthase complex, (b) OST complex, (c) Origin Recognition Complex and (d) snRNPs.


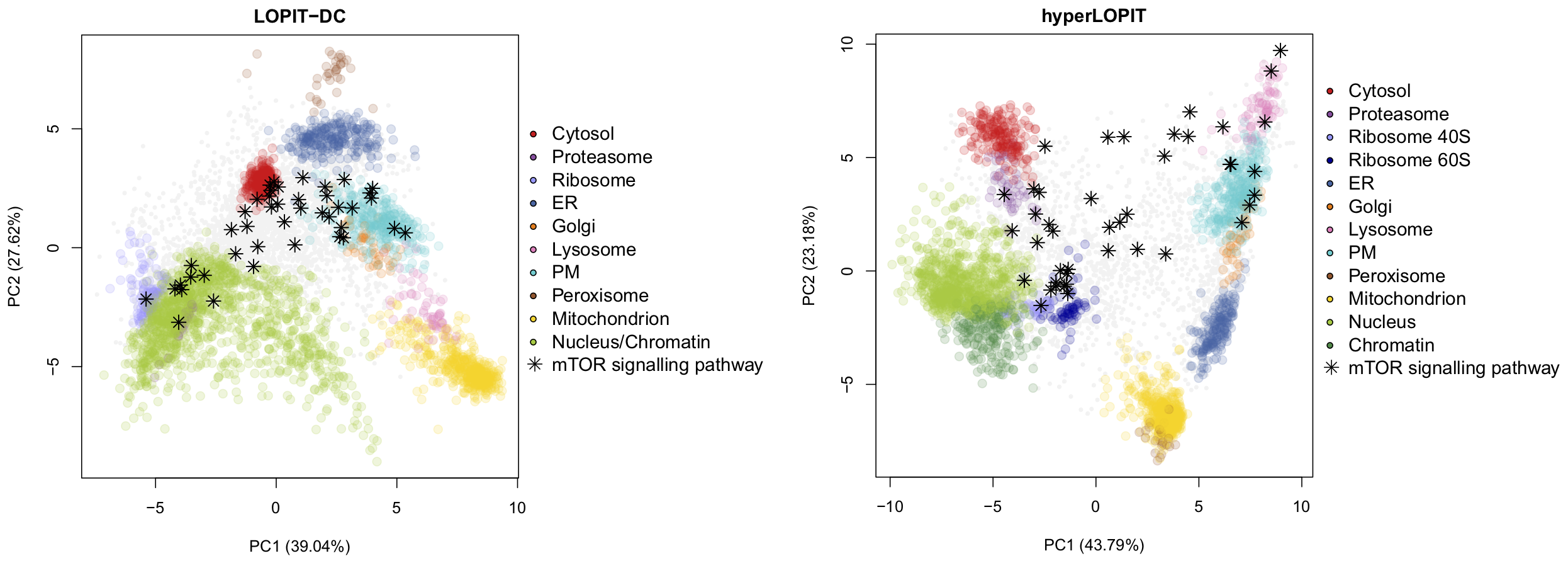


**Supplementary Figure 12 |** Proteins involved in mTOR signalling plotted upon the LOPIT-DC and hyperLOPIT datasets with assigned proteins.


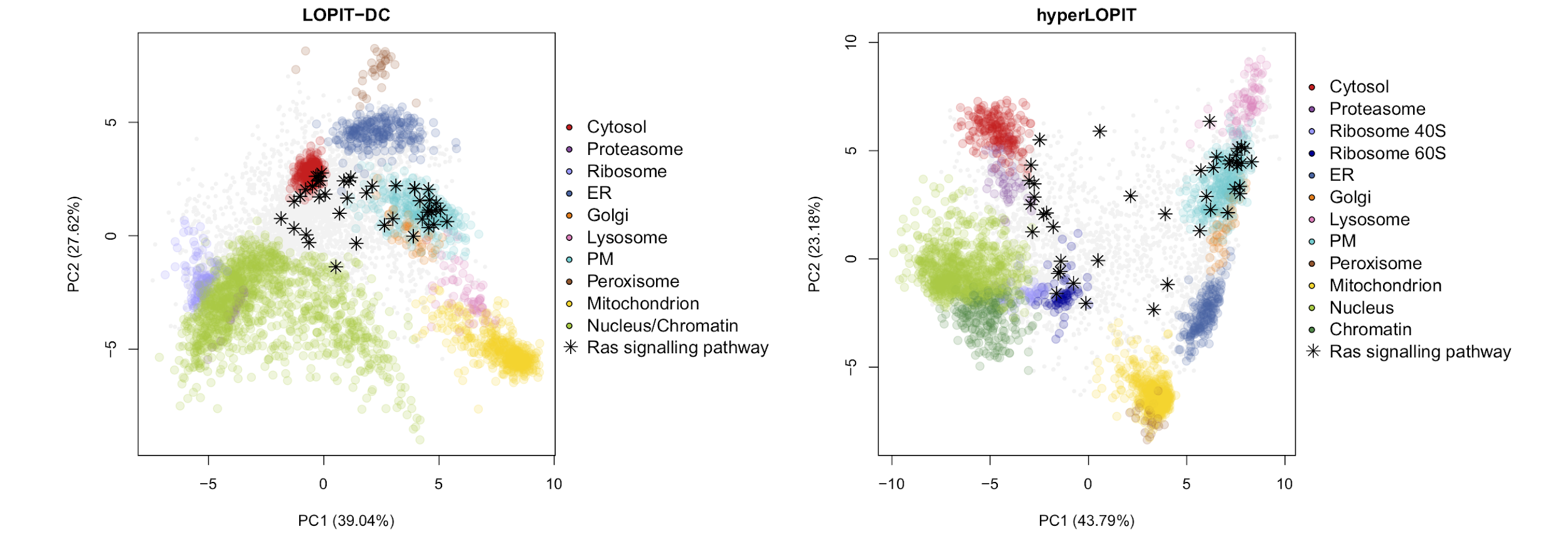


**Supplementary Figure 13 |** Proteins involved in Ras signalling plotted upon the LOPIT-DC and hyperLOPIT datasets with assigned proteins.


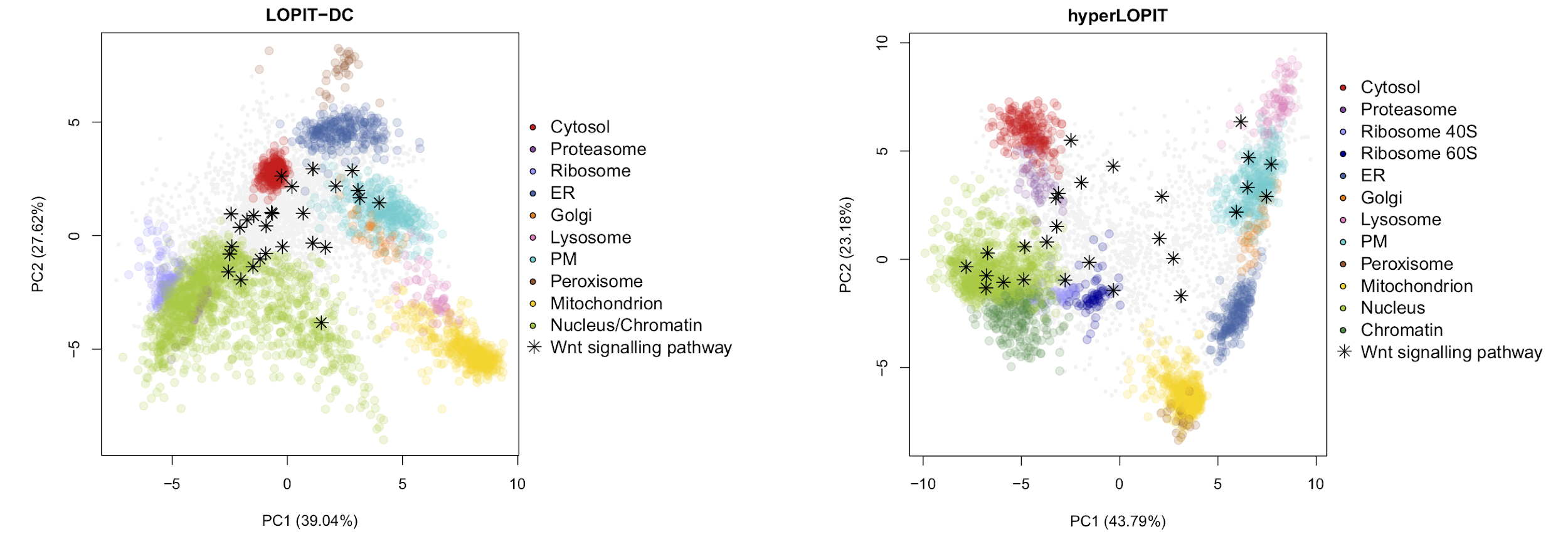


**Supplementary Figure 14 |** Proteins involved in Wnt signalling plotted upon the LOPIT-DC and hyperLOPIT datasets with assigned proteins.


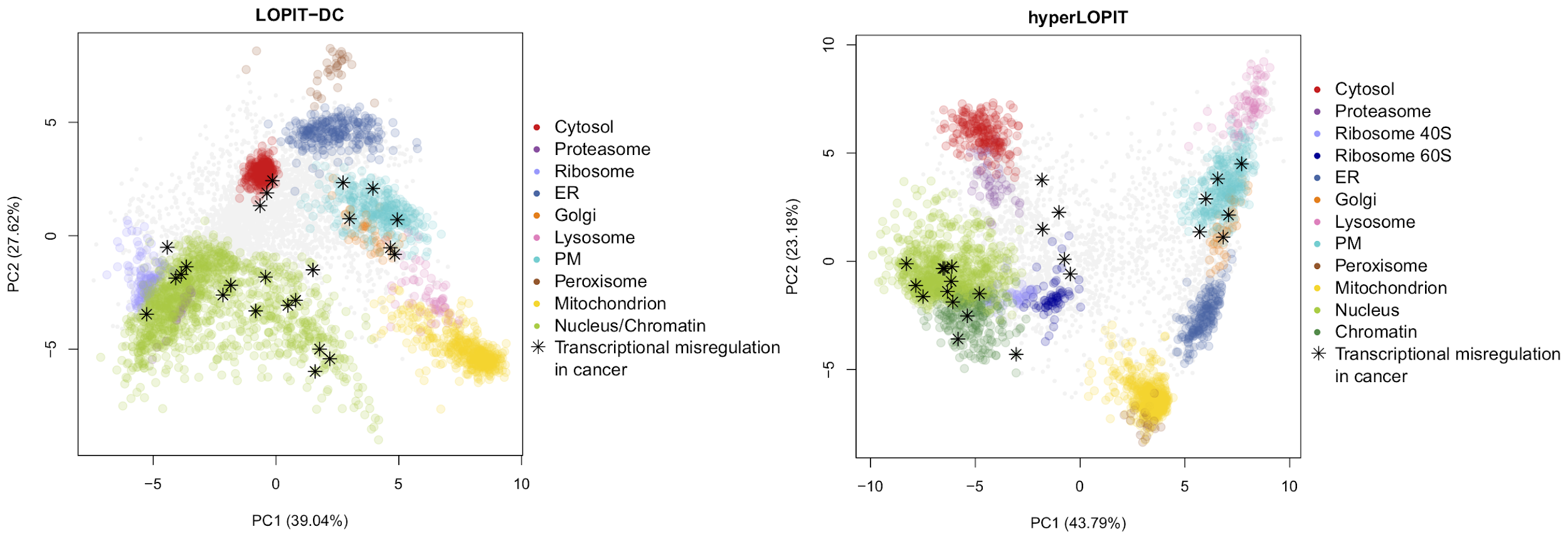


**Supplementary Figure 15 |** Proteins involved in transcriptional misregulation in cancer plotted upon the LOPIT-DC and hyperLOPIT datasets with assigned proteins.


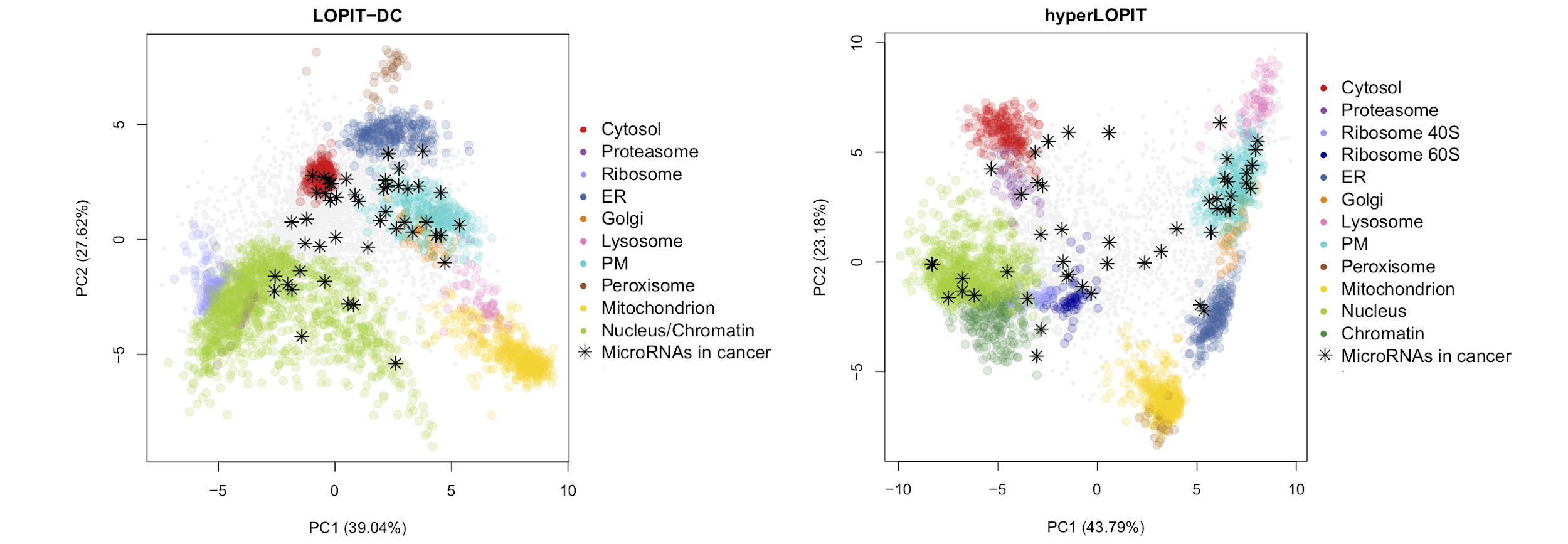


**Supplementary Figure 16 |** Proteins involved in microRNAs’ various roles in cancer plotted upon the LOPIT-DC and hyperLOPIT datasets with assigned proteins.

**
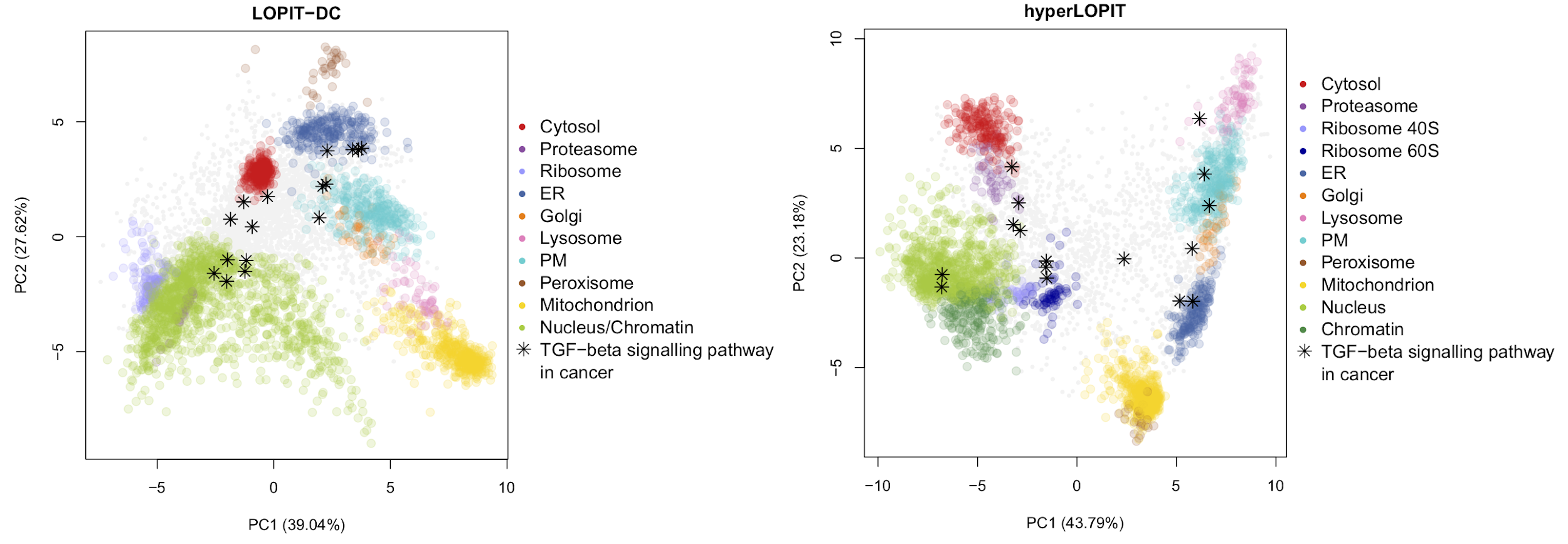
**

**Supplementary Figure 17 |** Proteins involved in the TGF-beta signalling pathway plotted upon the LOPIT-DC and hyperLOPIT datasets with assigned proteins.


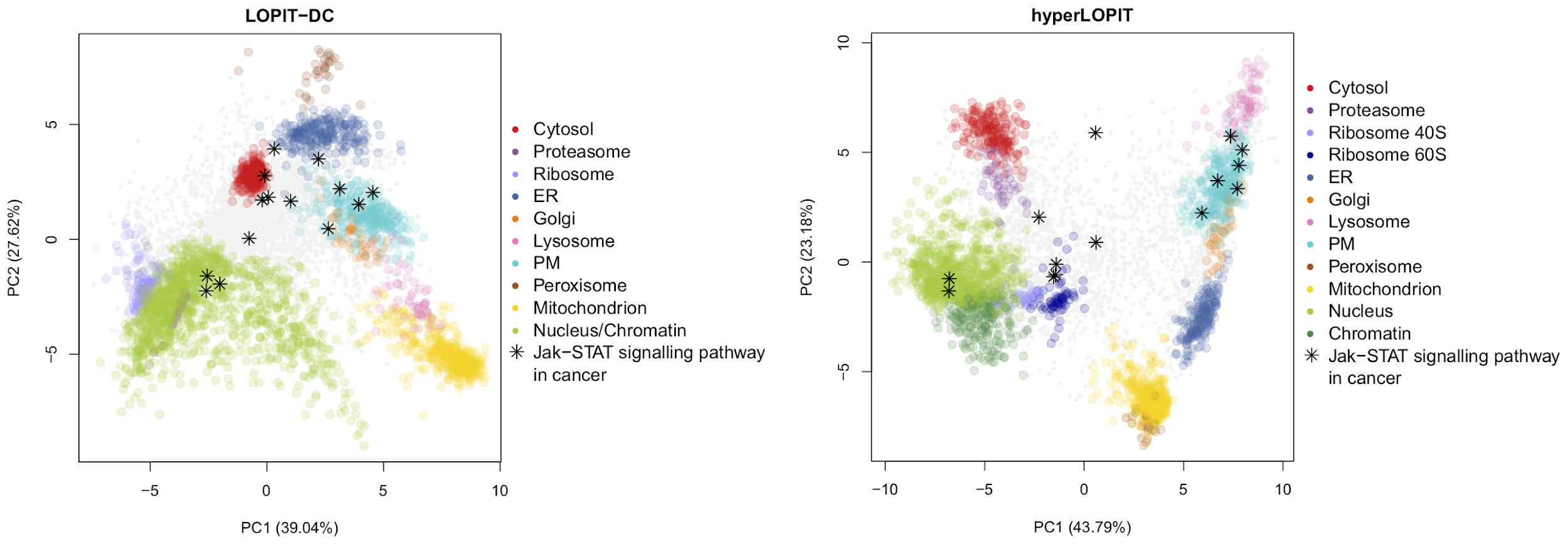


**Supplementary Figure 18 |** Proteins involved in the Jak-STAT signalling pathway plotted upon the LOPIT-DC and hyperLOPIT datasets with assigned proteins.


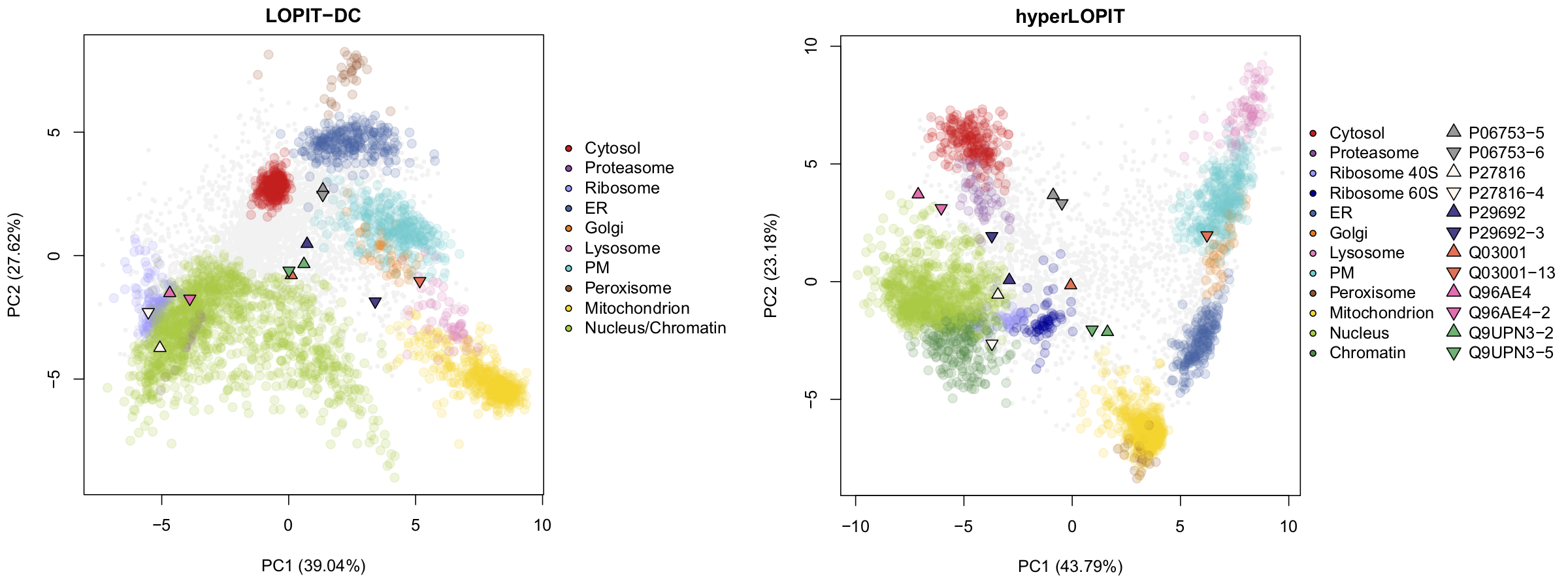


**Supplementary Figure 19 |** Six examples where two different isoforms of a protein were present in both the LOPIT-DC and hyperLOPIT datasets are plotted with assigned proteins.

**Supplementary Tables**

**Supplementary Table 1 |** Time-wise comparison between hyperLOPIT and LOPIT-DC.

| **Procedure** | **hyperLOPIT** | **LOPIT-DC** |
| --- | --- | --- |
| Cell number | 280 x 10^6 | 70 x 10^6* |
| Cell lysis | 5 h | 1-2 h |
| Crude membrane preparation | 3 h | - |
| Centrifugation | 8 h | 4-6 h |
| Fraction collection, membrane pelleting | 1 day | - |
| Membrane solubilisation | 2 h | 2 h |
| Reduction/alkylation | 2 h | 2 h |
| Tryptic digestion | 1 h + O/N | 1 h + O/N |
| Reducing peptides to dryness, TMT labelling and pooling, reducing to dryness | 1 day | 1 day |
| C18 clean-up, reducing peptides to dryness | 1 day | 1 day |
| RP-UPLC, reducing peptides to dryness | 1 day | 1 day |
| MS acquisition using SPS-MS^3^ | 3 days | 1.5 days |
| Data processing with Proteome Discoverer | < 2 days | < 1 day |
| Data visualisation, organelle marker assignment, classification (basic analysis) | 1 day | 1 day |

*can go as low as 50 x 10^6 cells to produce at least 50 µg in the lowest protein amount fraction

**Supplementary Table 2 |** Centrifugation speeds and times for the LOPIT-DC fractionation protocol.

| **Sample** | **Speed (x g)** | **Time (min)** |
| --- | --- | --- |
| Unlysed cell removal | 200 | 5 |
| Pellet 1 | 1000 | 10 |
| Pellet 2 | 3000 | 10 |
| Pellet 3 | 5000 | 10 |
| Pellet 4 | 9000 | 15 |
| Pellet 5 | 12000 | 15 |
| Pellet 6 | 15000 | 15 |
| Pellet 7 | 30000 | 20 |
| Pellet 8 | 79000 | 43 |
| Pellet 9 | 120000 | 45 |
| Supernatant | - | - |

**Supplementary Table 3 |** Identification results for the LOPIT-DC and hyperLOPIT datasets.

| 579 markers | **LOPIT-DC** | **hyperLOPIT** |
| --- | --- | --- |
| Protein groups | 9386 | 9558 |
| Merged proteins | 22944 | 23112 |
| Peptides | 100463 | 126368 |
| PSMs | 261857 | 447574 |
| Search inputs | 1424287 | 3057993 |
| Protein groups for quantitation after filtering and merging | 6837 | 4883 |

**Supplementary Table 4 |** Number of assigned proteins in the LOPIT-DC and hyperLOPIT data.

| **Subcellular location** | **LOPIT-DC** | **hyperLOPIT** |
| --- | --- | --- |
| Mitochondrion | 342 | 426 |
| Endoplasmic reticulum | 169 | 105 |
| Golgi apparatus | 12 | 7 |
| Plasma membrane | 259 | 267 |
| Lysosome | 26 | 40 |
| Peroxisome | 19 | 9 |
| Proteasome | 8 | 23 |
| Ribosome 40S | 66 | 23 |
| Ribosome 60S |  | 28 |
| Cytosol | 328 | 156 |
| Nucleus | 1183 | 765 |
| Chromatin |  | 189 |
