# Supplementary material for: Combining LOPIT with differential ultracentrifugation for high-resolution spatial proteomics
Source: Nat Commun. 2019 Jan 18;10:331. doi: 10.1038/s41467-018-08191-w (PMC6338729; doi:10.1038/s41467-018-08191-w)
Supplement: Supplementary file 3 — Description of Additional Supplementary Files [file 41467_2018_8191_MOESM3_ESM.docx]

**Description of Additional Supplementary Files**

**File Name**: Supplementary Data 1

**Description**: LOPIT-DC Quantitation Information. This file contains all quantitation data for the proteins identified using the LOPIT-DC workflow after initial processing using Proteome Discoverer v1.4, including protein accession number and description in UniProt, normalised TMT 10-plex reporter ion distribution for every protein in each replicate, whether a protein was used as an organelle marker during SVM-based subcellular localisation classification, initial SVM classification and SVM score for all proteins, and final SVM prediction for each protein by subcellular location after 5% FDR filtering.

**File Name:** Supplementary Data 2

**Description:** HyperLOPIT Quantitation Information. This file contains all quantitation data for the proteins identified using the hyperLOPIT workflow after initial processing using Proteome Discoverer v1.4, including protein accession number and description in UniProt, normalised TMT 10-plex reporter ion distribution for every protein in each replicate, whether a protein was used as an organelle marker during SVM-based subcellular localisation classification, initial SVM classification and SVM score for all proteins, and final SVM prediction for each protein by subcellular location after 5% FDR filtering.
