# Supplementary material for: Combining LOPIT with differential ultracentrifugation for high-resolution spatial proteomics
Source: Nat Commun. 2019 Jan 18;10:331. doi: 10.1038/s41467-018-08191-w (PMC6338729; doi:10.1038/s41467-018-08191-w)
Supplement: Supplementary file 6 — Reporting Summary [file 41467_2018_8191_MOESM6_ESM.pdf]

nature research

Corresponding author(s): Professor Kathryn S. Lilley

Last updated by author(s): Dec 14, 2018

Reporting Summary

Nature Research wishes to improve the reproducibility of the work that we publish. This form provides structure for consistency and transparency in reporting. For further information on Nature Research policies, see Authors & Referees and the Editorial Policy Checklist.

Statistics

For all statistical analyses, confirm that the following items are present in the figure legend, table legend, main text, or Methods section.

n/a	Confirmed	
☒	☐	The exact sample size (*n*) for each experimental group/condition, given as a discrete number and unit of measurement
☐	☒	A statement on whether measurements were taken from distinct samples or whether the same sample was measured repeatedly
☐	☒	The statistical test(s) used AND whether they are one- or two-sided<br>*Only common tests should be described solely by name; describe more complex techniques in the Methods section.*
☒	☐	A description of all covariates tested
☐	☒	A description of any assumptions or corrections, such as tests of normality and adjustment for multiple comparisons
☐	☒	A full description of the statistical parameters including central tendency (e.g. means) or other basic estimates (e.g. regression coefficient) AND variation (e.g. standard deviation) or associated estimates of uncertainty (e.g. confidence intervals)
☐	☒	For null hypothesis testing, the test statistic (e.g. *F*, *t*, *r*) with confidence intervals, effect sizes, degrees of freedom and *P* value noted<br>*Give P values as exact values whenever suitable.*
☒	☐	For Bayesian analysis, information on the choice of priors and Markov chain Monte Carlo settings
☒	☐	For hierarchical and complex designs, identification of the appropriate level for tests and full reporting of outcomes
☒	☐	Estimates of effect sizes (e.g. Cohen's *d*, Pearson's *r*), indicating how they were calculated

Our web collection on statistics for biologists contains articles on many of the points above.

Software and code

Policy information about availability of computer code

Data collection	Raw files from our experiments were processed with Proteome Discoverer v1.4 (Thermo Fisher Scientific) using the Mascot server v2.3.02 (Matrix Science). The SwissProt sequence database for Homo sapiens (canonical and isoform, 42,118 sequences, downloaded on 04/11/2016) was used along with common contaminants from the common Repository of Adventitious Proteins (cRAP) v1.0 (48 sequences, adapted from the Global Proteome Machine repository, https://www.thegpm.org/crap/). Precursor and fragment mass tolerances were set to 10 ppm and 0.6 Da, respectively. Trypsin was set as the enzyme of choice and a maximum of 2 missed cleavages were allowed. Static modifications were: methylthio (C), TMT6plex (N-term) and TMT6plex (K). Dynamic modifications were: oxidation (M) and deamidated (NQ). Percolator was used to assess the false discovery rate (FDR) and only high confidence peptides were retained. Additional data reduction filters were: peptide rank = 1 and ion score > 20.<br>Quantification at the MS3 level was performed within the Proteome Discoverer workflow using the centroid sum method and an integration tolerance of 2 mmu. Isotope impurity correction factors were applied. Each raw peptide-spectrum match (PSM) reporter intensity was then divided by the sum of all intensities for that PSM (sum normalisation). Protein grouping was carried out according to the minimum parsimony principle and the median of all sum-normalised PSM ratios belonging to each protein group was calculated as the protein group quantitation value.
Data analysis	Bioconductor packages pRoloc (version 1.21.9), pRolocdata (version 1.19.4) and MSnbase (version 2.6.1) were used to analyze the data. http://bioconductor.org/packages/release/bioc/html/pRoloc.html, https://bioconductor.org/packages/release/data/experiment/html/pRolocdata.html, https://bioconductor.org/packages/release/bioc/html/MSnbase.html. The data were analyzed according to the protocols published https://www.nature.com/articles/nprot.2017.026 and https://f1000research.com/articles/5-2926/v2. These packages provide vignettes detailing clearly how to install the packages into the R statistical computing language and the steps taken to perform data analysis. Version 3.7 of Bioconductor was used and version 3.5.1 of R was used.

For manuscripts utilizing custom algorithms or software that are central to the research but not yet described in published literature, software must be made available to editors/reviewers. We strongly encourage code deposition in a community repository (e.g. GitHub). See the Nature Research guidelines for submitting code & software for further information.

Data

Policy information about availability of data

All manuscripts must include a data availability statement. This statement should provide the following information, where applicable:

- Accession codes, unique identifiers, or web links for publicly available datasets
- A list of figures that have associated raw data
- A description of any restrictions on data availability

All protein-level datasets generated during this study are available in the R Bioconductor pRolocdata package (version >= 1.19.4) and can be viewed interactively via the pRolocGUI application (version 1.17.0) or directly online through the dedicated R Shiny apps ([https://proteome.shinyapps.io/lopitdc-u2os2018] and [https://proteome.shinyapps.io/hyperlopit-u2os2018]). All data have been deposited to the ProteomeXchange Consortium via the PRIDE partner repository with the dataset identifier PXD011254.

Field-specific reporting

Please select the one below that is the best fit for your research. If you are not sure, read the appropriate sections before making your selection.

☒ Life sciences ☐ Behavioural & social sciences ☐ Ecological, evolutionary & environmental sciences

For a reference copy of the document with all sections, see nature.com/documents/nr-reporting-summary-flat.pdf

Life sciences study design

All studies must disclose on these points even when the disclosure is negative.

Sample size	Sample size calculations were not relevant for this study.
Data exclusions	Only proteins with a full reporter ion series were retained. Additionally, three of the 60 TMT channels present in the final hyperLOPIT dataset possessed extremely low ion intensity profiles and were excluded from downstream data analysis to minimise background noise in the data. Finally, proteins identified as part of the common Repository of Adventitious Proteins (cRAP) were also removed for downstream analysis.
Replication	All data presented in this study were successfully replicated. Three replicates are presented for the LOPIT-DC data and three replicates for the hyperLOPIT dataset.
Randomization	Randomization was not relevant for this study.
Blinding	Blinding was not relevant to this study.

Reporting for specific materials, systems and methods

We require information from authors about some types of materials, experimental systems and methods used in many studies. Here, indicate whether each material, system or method listed is relevant to your study. If you are not sure if a list item applies to your research, read the appropriate section before selecting a response.

Materials & experimental systems

n/a	Involved in the study	
☒	☐	Antibodies
☐	☒	Eukaryotic cell lines
☒	☐	Palaeontology
☒	☐	Animals and other organisms
☒	☐	Human research participants
☒	☐	Clinical data

Methods

n/a	Involved in the study	
☒	☐	ChIP-seq
☒	☐	Flow cytometry
☒	☐	MRI-based neuroimaging

Eukaryotic cell lines

Policy information about cell lines

Cell line source(s)	The U-2 OS human osteosarcoma cell line (RRID: CVCL_0042) was a generous gift from Professor Emma Lundberg (SciLifeLab Stockholm and School of Biotechnology, KTH).
Authentication	The cell line used was not authenticated.
Mycoplasma contamination	The cell line used tested negative for mycoplasma contamination.

Commonly misidentified lines (See ICLAC register)	None of the published commonly misidentified cell lines were used in this study.
